# Myeloid differentiation primary response protein 88 (MyD88)-deficient dendritic cells exhibit a skewed cytokine response to BCG

**DOI:** 10.1186/s13104-019-4086-6

**Published:** 2019-01-23

**Authors:** Pawan Kumar, Sangeeta Bhaskar

**Affiliations:** 1 Present Address: Dept. of Preventive Oncology, Dr. B.R Ambedkar Institute Rotary Cancer Hospital, All India Institute of Medical Sciences, Ansari Nagar, New Delhi, 110029 India; 2 PDC-I, National Institute of Immunology, Aruna Asaf Ali Marg, New Delhi, 110067 India

**Keywords:** Dendritic cells, Cytokine secretion, Mycobacteria, MyD88, Tuberculosis, Immunity

## Abstract

**Objective:**

Macrophages and dendritic cells (DCs) play key role in the recognition of mycobacterial infection and mounting of antimycobacterial immunity. In case of macrophages, recognition of BCG and other mycobacteria has been attributed predominantly to MyD88-dependent singling. Interestingly, in previous study with bone marrow-derived DCs, we have shown that BCG promotes the survival of wild-type and MyD88^−/−^ cells to the comparable levels. In the present study, we further examined MyD88^−/−^ DC’s response to BCG.

**Results:**

Bone marrow-derived DCs from wild-type and MyD88^−/−^ mice were stimulated with BCG and analyzed for cytokine secretion. As expected, BCG-stimulated wild-type DCs produced significant amount of TNF-α and IL-12p40 in response to BCG. Interestingly, BCG-stimulated MyD88^−/−^ DCs were also found to secret significantly higher levels of TNF-α and IL-12p40, compared with unstimulated DCs. We further observed that wild-type DCs produced significant level of immunosuppressive cytokine IL-10 in response to BCG, whereas MyD88^−/−^ DCs secreted very low amount of IL-10 when stimulated with BCG. These findings demonstrated that MyD88^−/−^ DCs exhibit a skewed cytokine response to BCG.

## Introduction

Dendritic cells and macrophages are the antigen presenting cells involved in the recognition of BCG and other mycobacteria, and activation of antimycobacterial immunity [1]. They possess an array of pattern recognition receptors (PRRs), specialized in the recognition of molecular patterns present in pathogenic microbes. Toll-like receptors (TLRs) are one of the most prominent and most characterized trans-membrane PRRs. TLR engagement by mycobacteria has been shown to affect diverse immunological outcomes, including cytokine secretion, antigen presentation, cell survival, and cellular differentiation [1, 2].

MyD88 is an adapter protein, relaying TLR engagement signals across the plasma membrane. Defects in MyD88-dependent signalling have been shown to severely impair the innate immunity [3]. MyD88 deficiency in macrophages results in their hyporesponsiveness to BCG and other mycobacteria, characterized by diminished secretion of proinflammatory cytokines and inducible nitric oxide synthase (iNOS) [4, 5]. However, the role of MyD88-dependent signaling in DC response to mycobacteria remains poorly understood. A study by Fremond et al. [6] has demonstrated drastic reduction in IL-12 and IL-1β secretion by BCG-stimulated MyD88^−/−^ DCs, compared with wild-type DCs. On the other hand, heightened antimycobacterial T cell responses in MyD88^−/−^ mice suggests the potent DC responsiveness to the bacilli in these animals [7, 8].

In our previous study, we have shown that BCG promotes the survival of wild-type and MyD88^−/−^ DCs to the comparable levels [9]. In the present manuscript, we further examined the role of MyD88 in DC activation by BCG. Bone marrow-derived DCs from wild-type and MyD88^−/−^ mice were stimulated with BCG and analyzed for TNF-α, IL-12p40 and IL-10 secretion by ELISA. It was observed that both wild-type and MyD88^−/−^ DCs produced significant amounts of TNF-α and IL-12p40 in response to the bacilli. Further, we noticed that wild-type DCs, but not MyD88^−/−^ DCs secreted significant amount of IL-10 in response to BCG. These findings demonstrated a robust but skewed cytokine response of MyD88^−/−^ DCs to BCG.

## Main text

### Materials and methods

Wild-type and MyD88^−/−^ C57BL/6 mice of 6–8 weeks age were obtained from the animal house facility of the National Institute of Immunology, New Delhi. Mice were euthanized by CO_2_ asphyxiation method. DCs were derived from mouse bone-marrow cells by culturing them in the presence of GM-CSF (Peprotech Asia, Israel) as described previously [10]. Purity of dendritic cells, as determined on the basis of CD11c and MHCII expression by flow cytometry, was ~ 90%. BCG (strain Danish 1331, kindly provided by Prof. Anil Tyagi, University of Delhi South Campus, New Delhi, India) was cultured in 7H9 broth (BD Difco, India) supplemented with 10% albumin-dextrose-catalase (ADC) enrichment, 0.5% glycerol and 1% tween-80. Bacilli were cultured at 36 °C in static condition with intermittent manual shaking. DCs were plated in 24-well cell culture plate (1.5 × 10^6^ cells per well) and stimulated with BCG at the multiplicity of infection (MOI) of 10 as described previously [9]. Culture supernatants were collected after 24 h and analyzed for TNF-α, IL-12p40 and IL-10 using ELISA kits as described by the manufacturer (BD Biosciences). Data were analyzed by one way ANOVA (with Tukey’s comparison test applied post-analysis) using GraphPad Prism 5 software.

### Results

**Fig. 1:**
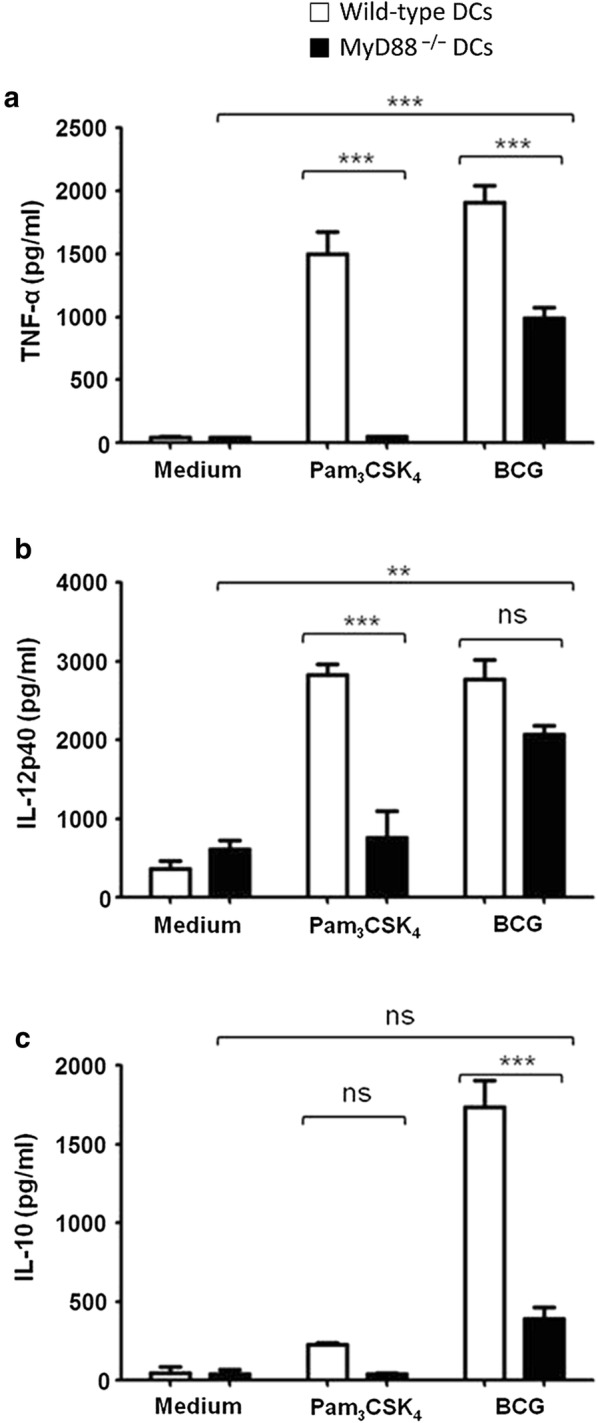
Cytokine secretion by MyD88^−/−^ dendritic cells (DCs) in response to BCG. Bone marrow-derived DCs from wild-type (open bars) and MyD88^−/−^ mice (filled bars) were stimulated with BCG at MOI of 10. Culture supernatants were harvested after 24 h and analyzed for TNF-α, IL-12p40. Significant levels of TNF-α and IL-12p40 were produced by BCG-stimulated wild-type and MyD88^−/−^ DCs, compared with unstimulated DCs (**a**, **b**). Culture supernatants were also analyzed for IL-10 levels. Significant amount of IL-10 was secreted by BCG-stimulated wild-type, but not by MyD88^−/−^ DCs (**c**). Synthetic TLR2 ligand Pam_3_CSK_4_ (100 ng/ml) was used as an experimental control. Data shown are mean ± SEM of three independent experiments. **p < 0.01, ***p < 0.001. *ns* not significant

Culture supernatants from BCG-stimulated wild-type and MyD88^−/−^ DC were analyzed for proinflammatory cytokines TNF-α and IL-12p40. As expected, wild-type DCs produced significant amounts of TNF-α and IL-12p40 in response to BCG, compared with unstimulated DCs (Fig. 1a, b). Interestingly, BCG-stimulated MyD88^−/−^ DCs also produced significant levels of TNF-α and IL-12p40, compared with unstimulated MyD88^−/−^ DCs. Levels of IL-12p40 secreted by BCG-stimulated wild-type and MyD88^−/−^ DCs were comparable, whereas TNF-α secretion by BCG-stimulated wild-type DCs was significantly higher, compared with that produced BCG-stimulated MyD88^−/−^ DCs (Fig. 1a, b). We also examined IL-10 secretion by BCG-stimulated wild-type and MyD88^−/−^ DCs. IL-10 is an immunosuppressive cytokine involved in controlling the collateral damage to surrounding tissue during immune reaction [11]. It was observed that BCG-stimulated wild-type DCs produced significant amount of IL-10. However, MyD88^−/−^ DCs failed to produce a significant level of IL-10 in response to BCG (Fig. 1c). Synthetic TLR2 ligand Pam_3_CSK_4_ was used as an experimental control.

### Discussion

The present study was undertaken to gain insight into our previous observations, wherein BCG was found to promote survival in both wild-type and MyD88^−/−^ DCs [9]. In keeping with these observations, the present study demonstrates the potent responsiveness of MyD88^−/−^ DCs to BCG. Similar to wild-type DCs, MyD88^−/−^ DCs produced significant levels of IL-12p40 and TNF-α in response to BCG. IL-12 promotes differentiation of naïve T cells into T_H_1 cells and stimulates IFN-gamma production by T lymphocytes [1, 12]. TNF-α is involved in macrophage activation, inflammation and other immunological phenomena [1, 12]. In agreement with production of inflammatory cytokines by BCG-stimulated MyD88^−/−^ DCs, MyD88-deficient mice have been shown to mount strong T cell responses to mycobacterial infection [7].

DCs, being the key antigen presenting cells, exert control over T cell responses. Depending on the nature of antigen, DCs can induce normal or hyperactive T cell responses or can induce T cell anergy. Different outcomes of antigen presentation by DCs rely on delicate balance of proinflammatory (i.e. IL-12, IL-17, TNF-α) and anti-inflammatory (i.e. IL-10, TGF-β) cytokines. Interestingly, our results showed that in contrast to wild-type DCs, which produced both pro- and anti-inflammatory cytokines, MyD88^−/−^ DCs secreted drastically reduced level of IL-10 in response to BCG. IL-10 is an immunosuppressive cytokine (involved in the peripheral immune tolerance); IL-10-deficient mice have been shown to develop a variety of inflammatory and autoimmune conditions [11].

Skewed cytokine response of BCG-stimulated MyD88^−/−^ DCs provides an important insight into aggravated immune response and high mortality in *M. tuberculosis* (*Mtb*)-infected MyD88^−/−^ mice [7, 8]. In the absence of IL-10, predominant activity of proinflammatory cytokines drives the T cell response and inflammatory reaction to the pathologically intense levels, which could lead to lung tissue damage and death. What molecular mechanisms are involved in mycobacterial recognition by MyD88^−/−^ DCs, however, remains a key question. DCs are known to express a large repertoire of PRRs and we are further examining different PRRs for their involvement in MyD88^−/−^ DC activation by BCG. Taken together, our findings demonstrate the potent response of MyD88^−/−^ DCs to BCG and suggest a putative mechanism for overly active antimycobacterial immune response in *Mtb*-infected MyD88^−/−^ mice.

## Limitations

DCs are heterogeneous cell population. Multiple DC subsets (e.g. lymphoid, myeloid, plasmacytoid, langerhans cells) with significant variations in their functions have been defined in mice and humans [13]. The present study has been carried out using bone marrow-derived DCs from wild-type and MyD88^−/−^ mice. Therefore, results presented in this manuscript are specifically valid for these cells.

## Abbreviations

DC: dendritic cell
PRRs: pattern recognition receptors
TLR: toll-like receptor
APC: antigen-presenting cells
BCG: bacillus Calmette-Guerin
*Mtb*: *Mycobacterium tuberculosis*
